# Genomic Cues From Beta-Coronaviruses and Mammalian Hosts Sheds Light on Probable Origins and Infectivity of SARS-CoV-2 Causing COVID-19

**DOI:** 10.3389/fgene.2020.00902

**Published:** 2020-08-31

**Authors:** Charles A. Narh

**Affiliations:** ^1^Life Sciences, Burnet Institute for Medical Research, Melbourne, VIC, Australia; ^2^Department of Medicine, University of Melbourne, Melbourne, VIC, Australia; ^3^Department of Infectious Diseases, Monash University, Melbourne, VIC, Australia

**Keywords:** COVID-19, SARS-CoV-2, spike-glycoprotein, furin-protease, origin, pangolin, bat, inflammasome

## Introduction

**Figure 1:**
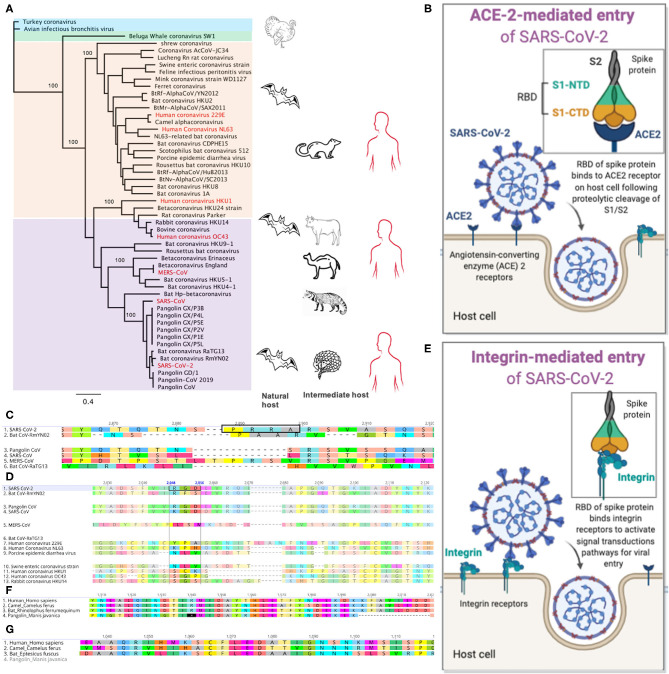
Genomic signatures from beta-coronavirus and their mammalian hosts associated with SARS-CoV-2 infection and pathogenesis in humans. **(A)** Phylogenetic relatedness of coronaviruses causing infections in mammals. The tree was drawn using whole genome sequences of beta- (purple), alpha- (peach), gamma- (blue), and delta- (green) coronaviruses. Infections in humans are shown in red. Bats are natural reservoirs of alpha- and beta-coronaviruses but their direct role in the transmission of these viruses to humans remains a puzzle. The closest relative to SARS-CoV-2 is the bat coronavirus RaTG13, which lacks key amino acid residues in its spike protein that binds human ACE2 receptors. The recently discovered Pangolin-CoV 2019 is the third closest relative to SARS-CoV-2 and it has nearly identical amino acid residues in its spike protein as the SARS-CoV-2 S, suggesting possible infection in humans. SARS-CoV-2 may have evolved from a series of recombination events on the genetic background of bat-CoV-like and/or pangolin-CoV-like ancestor. The pangolin may be a possible intermediate host of SARS-CoV-2-like coronaviruses. Palm civets and camels, respectively, are intermediate hosts for SARS-CoV and MERS-CoV, respectively. **(B)** SARS-CoV-2 entry into host cell through binding of its receptor binding domain (RBD) in the spike (S) protein to the angiotensin-converting enzyme 2 (ACE2) receptor. The S1 subunit is composed ot the N-terminal domain (NTD) and C-terminal domain (CTD). **(C)** Alignment of the spike protein from five beta-coronaviruses. The furin cleavage site with amino acid motif “PRRA” (highlighted in the black box) is only present in SARS-CoV-2. **(D)** The integrin-binding motif, “RGD” is present in SARS-CoV-2 and pangolin-CoV. **(E)** Plausible entry of host cell via binding of the RGD amino acid motif in SARS-CoV-2 RBD to integrin protein within the cell membrane. **(F)** Alignment of the IFIH1 genes in humans, camel, bat and pangolin. IFIH1is a pseudogene in pangolin—presence of premature stop codons (^*^) and deletions. **(G)** The ZBP1 gene is absent in the pangolin.

Coronaviruses (CoV) including SARS-CoV and MERS-CoV were responsible for two major pneumonia outbreaks—Severe Acute Respiratory Syndrome (SARS, outbreak in 2003) and Middle East Respiratory Syndrome (MERS, outbreak in 2012) (WHO, 2003; Wu et al., 2020). In humans, CoV infections including CoV-229E, -NL63, -OC43, and -HKU1 (Figure 1A) are seasonal and cause mild upper and lower respiratory tract disease with clinical presentations similar to the flu (Koetz et al., 2006; Gaunt et al., 2010). In December 2019, a novel CoV, now officially named SARS-CoV-2 emerged in Wuhan, China. SARS-CoV-2, a beta-coronavirus (Figure 1A), causes coronavirus disease 2019, simply called COVID-19 (Chan et al., 2020; Zhu et al., 2020). The pathogenesis and clinical outcome of SARS-CoV-2 infection is similar to SARS with fever, cough, and shortness of breath being the most commonly reported symptoms (Mcarthur et al., 2020). SARS-CoV-2 is now pandemic and has infected more than 12 million people and caused more than half a million deaths globally.

The high infectivity and transmissibility of SARS-CoV-2 in comparison to SARS-CoV and MERS-CoV (Petrosillo et al., 2020) have raised several questions—what is the origin and evolution of the virus? Did SARS-CoV-2 evolved from a beta-coronavirus? Did it evolved in an intermediate host before spilling over into humans? when and how would this have happened and are there evolutionary cues to direct researchers as to where to look for answers? The first cluster of COVID-19 index cases in Wuhan were associated with the Huanan Seafood Wholesale Market in the Hubei Province of China (Lu et al., 2020). There are reports that some wild and farmed animals including birds, reptiles, and mammals are sometimes slaughtered in the market and their meat is sold for food (Nationalgeography, 2020). There is no direct evidence to source-track the first SARS-CoV-2 infections in humans although SARS-CoV-2 genetic material was detected in environmental samples collected from the Wuhan Seafood Market (WHO, 2020).

Mammals including bats and pangolins are natural reservoirs of SARS-CoV-2-related viruses and may likely be sources of the first SARS-CoV-2 infections in humans (Figure 1A) (Ye et al., 2020). The association of the first cases of SARS-CoV-2 infection and exposure to the Seafood Market in Wuhan led some researchers to propose that SARS-CoV-2 originated from pangolins and bats, which are sources of food in Southern China. Additionally, their products are used for Chinese medicines (Li et al., 2005; Zhang and Holmes, 2020). These mammals including rodents and camels have been reported as natural or intermediate hosts of beta-coronaviruses (Figure 1A). During the SARS and MERS outbreak, palm civets, and camels were reported as intermediate hosts of SARS-CoV and MERS-CoV, respectively (Figure 1A) (De Wit et al., 2016). CoV-OC43 and CoV-HKU1, and CoV-229E and CoV-NL63 are believed to have jumped from rodents and bats, respectively, to humans (Ye et al., 2020). Although the exact source of infection remains unknown, recent findings from genomic studies have provided useful cues to suggest a probable origin of SARS-CoV-2.

## SARS-CoV-2 Is Phylogenetically Related to Beta-Coronaviruses That Infect Bats and Pangolins

Phylogenetically, the closest relatives to SARS-CoV-2, at the whole genome level, are the bat RaTG13 CoV (isolated from *Rhinolopus affinis*) and bat RmYN02 CoV (isolated from *Rhinolopus mayalanus*) with nucleotide similarities of 97 and 93.3%, respectively (Zhou et al., 2020), suggesting bats as the most likely natural reservoirs. Yet, the exact role that bats played in the emergence of SARS-CoV-2 remains a puzzle with no reported case of bat-to-human transmission. Could it be that an intermediate host was involved in the transmission of SARS-CoV-2 to humans? Possibly an animal that naturally carries the infection without developing clinical disease; one that humans come into contact with? Is it possible that other mammals such as pangolins served as intermediate hosts in the transmission of SARS-CoV-2 to humans?

Recently, metagenomic sequencing of samples collected from Malayan pangolins (*Manis Javanica*) in Southern China identified pangolin-associated coronaviruses that belonged to two sub-lineages of SARS-CoV-2-related coronaviruses (Lam et al., 2020). This novel pangolin-CoV is the third closest relative of SARS-CoV-2 with a 92.4% nucleotide sequence similarity at the genomic level (Figure 1A). Whether this finding is sufficient to implicate pangolins as natural or intermediate hosts of SARS-CoV-2-like coronaviruses needs to be investigated with large scale sampling of pangolin populations.

## SARS-CoV-2 Spike Glycoprotein Harbors Furin-Like and Integrin-Binding Recognition Sequences That May Increase Its Infectivity in Humans

The surface glycoprotein or spike protein (S) of beta-coronaviruses is integral to the establishment of infections in humans; it is a target of antibody-mediated immunity. The S protein is composed of the S1 and S2 subunits, which are cleaved during entry into the cell (Figure 1B). The S1 peptide facilitates attachment to the host cell; it utilizes the receptor binding domain (RBD) to bind the human angiotensin-converting enzyme 2 (hACE2) receptor (Hoffmann et al., 2020a). The S2 subunit mediates fusion into the host cell membrane and by endocytosis, it gains entry into the host cells (Figure 1B). Both SARS-CoV-2 and SARS-CoV utilize the ACE2 for viral entry and share 76% amino acid sequence identity in their S protein. The SARS-CoV-2 S and bat RaTG13 S share 98% amino acid sequence in the ectodomain but differ substantially with ~80% substitutions in the RBD (Wrobel et al., 2020); Prominent among these substitutions is the presence of a furin-like amino acid motif, “PRRA” (residues 682 and 685) between the S1 and S2 subunits of SARS-CoV-2 S (Figure 1C) (Coutard et al., 2020; Wang Q. et al., 2020). The RmYN02 CoV harbors a novel insertion sequence with the amino acid motif, “PAA” but its role in cell entry is not known (Figure 1C) (Zhang and Holmes, 2020; Zhou et al., 2020). In some viral infections such as HIV, influenza, and dengue, cleavage of the furin-like motif in the viral envelope protein, mediated by host furin proteases, facilitates endocytosis of the virus into host cells following cleavage at the S1/S2 furin site (Braun and Sauter, 2019).

Prior to cell entry, the SARS-CoV-2 S protein is cleaved by proprotein convertase furin at the S1/S2 site and by the transmembrane serine protease, TMPRSS2, at the S2 site (Millet and Whittaker, 2015; Hoffmann et al., 2020b). The furin-like motif is absent in the RBD of other beta-coronaviruses including SARS-CoV (Figure 1C)—its S protein is cleaved by trypsin, which is expressed in the respiratory tract (Hoffmann et al., 2018). Introduction of a furin motif into the RBD of SARS-CoV enhanced fusion but not its infectivity to the host cell (Follis et al., 2006), suggesting that additional mechanisms besides the furin-cleavage and ACE2-mediated entry may be playing a role in the higher infectivity of SARS-CoV-2 compared to SARS-CoV (Wrapp et al., 2020).

Plausibly, SARS-CoV-2 entry into host cells via a non-ACE2-mediated pathways including integrin-mediated entry may contribute to its higher infectivity than other beta-coronaviruses including SARS-CoV. Integrins are a family of transmembrane heterodimeric receptors that bind extracellular matrix ligands, cell-surface and soluble ligands, and activate signal transduction pathways (Takada et al., 2007). Integrins such as α5β1, αVβ1, αVβ3, αVβ5, αVβ6, αVβ8, and αIIbβ3 bind the “RGD” amino acid motif (Figure 1D) within viral envelope proteins. This binding activates phosphatidylinositol 3-kinase (PI3K) and mitogen-activating kinase (MAPK) signaling to enable endocytosis of the uncoated virus into the host cell (Takada et al., 2007). The integrin-mediated pathway (Figure 1E) is exploited by viruses such as ebolavirus, rotavirus, metapneumovirus, and Epstein-Bar virus (Schornberg et al., 2009; Hussein et al., 2015) and may likewise be utilized by SARS-CoV-2; it harbors the RGD motif in the RBD (residues 403–405) (Figure 1D). These residues are reportedly exposed following S1/S2 cleavage (Sigrist et al., 2020; Wrobel et al., 2020), which suggest that SARS-CoV-2 may be exploiting both ACE2 and integrin-mediated pathways to efficiently infect host cells. Integrins are ubiquitous and located on nucleated cells (Lowell and Mayadas, 2012), which may explain why SARS-CoV-2 can establish extrapulmonary, enteric and possibly, systemic infections (Wang W. et al., 2020; Zang et al., 2020). This extensive cellular tropism may explain its higher infectivity compared to SARS-CoV and likely play a role in the pathogenesis and the typical spectrum of clinical disease associated with COVID-19 (Mcarthur et al., 2020).

## SARS-CoV-2 May Have Evolved Naturally on the Genetic Background of Beta-Coronavirus Lineages Infecting Bats and Pangolins

Insertions and deletions (indels) of nucleotides within coronavirus genomes may constitute a series of recombination and/or natural events that increases their ability to infect and be transmitted between susceptible host and “jump” across species (Zhou et al., 2020). The novel pangolin-CoV S has 97.4% amino acid similarity to SARS-CoV-2 S and retains the five key amino acids—L458, F488, Q495, N503, and Y507 in the RBD, that interact with ACE2 (Lam et al., 2020; Zhang T. et al., 2020; Zhou et al., 2020). It also possesses the RGD integrin-binding motif (residues 399–401) (Figure 1D), suggesting similar cell invasion pathways in its pangolin host similar to SARS-CoV-2 in its human host. These recent insights into the novel pangolin-CoV and SARS-CoV-2 genomes also revealed putative recombination sites in SARS-CoV-2 genes including ORF1a and ORF8, which likely originated from the bat-CoV-like and SARS-CoV-like genomes (Lam et al., 2020; Li et al., 2020; Liu P. et al., 2020; Zhang T. et al., 2020). Chances of recombination is high when bats and/or mammalian intermediate hosts are co-infected with two or more coronaviruses with distinct genomes (Ye et al., 2020).

## No Evidence to Support Theories That SARS-CoV-2 Was Artificially Generated and/or Deliberately Released Into the Human Population

Theories of SARS-CoV-2 being a recombinant coronavirus that was genetically engineered from an existing beta-coronavirus or a novel virus that was generated *de novo* from the laboratory have not been supported with credible evidence. Comparative genomic analysis with chimeric CoVs that were generated from reverse genetic experiments using bat-CoV indicated that SARS-CoV-2 was highly divergent (>5000 nucleotides) from these strains (Liu S.-L. et al., 2020). Furthermore, there is no plausible evidence to support a deliberate and targeted introgression of beta-CoV-like genetic elements into a beta-CoV construct to generate SARS-CoV-2 (Liu S.-L. et al., 2020). Rather, the pattern of gene synteny between SARS-CoV-2 and other beta-CoVs suggest that SARS-CoV-2 evolved naturally (Andersen et al., 2020; Liu S.-L. et al., 2020). While it is unlikely that SARS-CoV-2 was artificially generated and deliberately released into the environment from a laboratory setting, a recent study demonstrated that a viable SARS-CoV-2 can be genetically engineered *de novo* (Thi Nhu Tha et al., 2020). Investigations into the origin of SARS-CoV-2 are urgently needed to help inform public health control and surveillance; The nature of these enquiries need international backings and should not be politicized (CNN, 2020; The Conversation, 2020; WHO, 2020).

## Bats and Pangolins May Have Evolved Mechanisms to Tolerate Coronavirus Infections Without Developing Clinical Illness

Bats have evolved innate and adaptive mechanisms to tolerate numerous viral infections including the coronaviruses that caused SARS and MERS. A few studies have suggested that antiviral responses in bats have been dampened due, in part, to the loss of a family of proteins called PYHIN proteins (Zhang et al., 2013; Ahn et al., 2016; Kumar et al., 2019). The PYHIN proteins function as immune sensors and activators of the inflammasome and apoptosis pathways in response to intracellular self or foreign nucleic acids (Bertin and Distefano, 2000; Banerjee et al., 2020). Comparative genomic analysis showed that the PYHIN gene family is absent across 10 bat species (Ahn et al., 2016) and the NLRP3 activation system of pro-inflammatory cytokines such as interleukin-1 beta (IL1-beta) is dampened in bats (Banerjee et al., 2020). The loss of the PYHIN gene family and the dampened immune response in bats may be an evolutionary adaption to coexist with numerous viral infections. By not killing the host, coronaviruses are successfully transmitted between susceptible mammals including humans. Transmission of coronaviruses from bats to humans has not been reported but it may be possible either directly through contact with infected bats and their products or indirectly when humans come into contact with bat CoV-contaminated environments, flowers, and fruits (Calisher et al., 2006; WHO, 2020).

Two antiviral genes—IFIH1 and ZBP1 in mammals involved in RNA sensing and activation of the inflammasome in response to viral infections are absent in pangolins. Pangolins lack a functional IFIH1 (Figure 1F), a protein that binds double-stranded RNA to mediate expression of interferon and to activate the inflammasome; IFIH1 is a pseudogene in pangolins (Fischer et al., 2020). Similarly, ZBP1 (Figure 1G), a protein that binds left-handed double-stranded RNA or DNA to trigger necroptosis and inflammation is absent in the Malayan pangolin (Fischer et al., 2020). Apparently, this loss of function is not detrimental to their survival; they have other intact genes including the retinoic acid-inducible gene I (RIG-I) that is involved in antiviral defense. Although this evolutionary insight is not sufficient to explain coronavirus infection in pangolins, it suggests that pangolins evolved to tolerate coronavirus infections without developing clinical illness. This biological plausibility may be extended to other mammals including rodents that have been identified as natural reservoirs of coronaviruses (Tsoleridis et al., 2016; Ge et al., 2017).

## SARS-CoV-2 Evolution and Selection After Jumping Into Humans

Genomic analysis of SARS-CoV-2 infections collected across the world indicated that the virus had mutated multiple times during the course of the pandemic. Two major mutant lineages of SARS-CoV-2 were associated with pathogenesis and transmissibility (Zhang L. et al., 2020). In particular, a mutation, D614G in the spike protein has been associated with higher infectivity compared to the wildtype, D614. SARS-CoV-2 strains with this mutation are now predominant globally (Korber et al., 2020). Several other mutations in the ORF1ab replicase genes including the RNA-dependent RNA polymerase have been detected in European, North American, and Asian strains of the virus (Pachetti et al., 2020). Nearly 80% of mutations detected in more than 7,000 SARS-CoV-2 genomes were non-synonymous and recurred frequently (Van Dorp et al., 2020), which may suggest that the virus is undergoing positive selection. These findings indicate that SARS-CoV-2 is patho-adapting to its human host, possibly to cause asymptomatic or mild disease in the majority of human infections while increasing its infectivity and transmissibility between susceptible host. Thus, it is important that evolutionary investigations include phenotypic characterizations of new SARS-CoV-2 genetic variants in circulation.

## Conclusion

It is possible that beta-coronaviruses such as the SARS-CoVs, pangolin-CoV, RaTG13 CoV, and bat RmYN02 CoV detected in humans, Malayan pangolins and bats, respectively, also cause infections in other mammals including livestock and wild rodents sold for food. These infections may be asymptomatic. Without causing overt disease and killing their host, these infections would be sufficiently transmitted from the natural or intermediate hosts to susceptible host including humans. Transmission could be through direct contact with the infected animal or their products. The acquisition of furin and integrin-binding recognition sequences in SARS-CoV-2 may have occurred pre- or post-spillover into humans. In addition to this, recombination and natural selection in an intermediate host together with other unknown evolutionary events that might have occurred on the genetic background of a bat-CoV-like and/or a pangolin-CoV-like ancestor may have increased SARS-CoV-2 zoonotic potential and infectivity to humans. The question of when these events happened warrants investigations and will be pivotal to help us understand how coronaviruses evolve across different species lines. The cross-species infectivity of coronaviruses and their zoonotic capabilities are great threats to public health and underscores the need for continuous surveillance, both in humans and other mammals.

## Author Contributions

CN designed, drafted, and submitted the manuscript.

## Conflict of Interest

The author declares that the research was conducted in the absence of any commercial or financial relationships that could be construed as a potential conflict of interest.
